# Hydrocephalus: Molecular and Neuroimaging Biomarkers in Diagnosis and Management

**DOI:** 10.3390/biomedicines13071511

**Published:** 2025-06-20

**Authors:** Andrada-Iasmina Roşu, Diana Andrei, Laura Andreea Ghenciu, Sorin Lucian Bolintineanu

**Affiliations:** 1 Doctoral School, University of Medicine and Pharmacy “Victor Babes”, Eftimie Murgu Square No. 2, 300041 Timisoara, Romania; iasmina.rosu@umft.ro; 2 Department of Anesthesia and Intensive Care, University of Medicine and Pharmacy “Victor Babes”, Eftimie Murgu Square 2, 300041 Timisoara, Romania; 3 Department of Balneology, Medical Rehabilitation and Rheumatology, University of Medicine and Pharmacy “Victor Babes”, 300041 Timisoara, Romania; 4 Department of Functional Sciences, Discipline of Pathophysiology, University of Medicine and Pharmacy “Victor Babes”, 300041 Timisoara, Romania; bolintineanu.laura@umft.ro; 5 Center for Translational Research and Systems Medicine, University of Medicine and Pharmacy “Victor Babes”, 300041 Timisoara, Romania; 6 Department of Anatomy and Embriology, University of Medicine and Pharmacy “Victor Babes”, 300041 Timisoara, Romania; s.bolintineanu@umft.ro

**Keywords:** hydrocephalus, neurological disease, normal pressure hydrocephalus, CSF, MRI, CT, biomarkers, molecular markers, neuroimaging markers, genetic markers

## Abstract

Hydrocephalus is a complex neurological condition marked by abnormal cerebrospinal fluid (CSF) accumulation, often leading to elevated intracranial pressure and structural brain damage. Despite advances in surgical treatment, diagnostic precision and prognosis remain challenging, especially in idiopathic normal pressure hydrocephalus (iNPH). This narrative review aims to synthesize the current knowledge regarding molecular and neuroimaging biomarkers that hold diagnostic, prognostic, and therapeutic significance in hydrocephalus. A comprehensive literature search was conducted across PubMed, Scopus, Web of Science, and Google Scholar. The inclusion criteria encompassed peer-reviewed studies involving congenital or acquired hydrocephalus and reporting on mechanistic, diagnostic, or monitoring biomarkers. Both established and emerging biomarkers were included, and preclinical findings were considered when translational relevance was apparent. The review highlights a broad spectrum of molecular markers including aquaporins, vascular endothelial growth factor, neurofilaments, glial fibrillary acidic protein, matrix metalloproteinases, and neuroinflammatory markers. The genetic markers associated with ciliogenesis also show promise in subtyping disease. Parallel to molecular advances, neuroimaging techniques, ranging from classic markers like Evans’ index to advanced modalities such as diffusion tensor imaging (DTI), arterial spin labeling (ASL), and glymphatic MRI, provide functional perspectives on hydrocephalus diagnosis and management, while artificial intelligence may further enhance diagnostic algorithms. Molecular and imaging markers could not only increase diagnostic confidence, but also provide information on disease causes and progression. As research progresses, merging various methodologies may result in more accurate diagnoses.

## 1. Introduction

Hydrocephalus is a neurological disorder characterized by an abnormal accumulation of cerebrospinal fluid (CSF) within the ventricular system of the brain, leading to increased intracranial pressure and potential damage to brain structures. Normal pressure hydrocephalus (NPH) was first described in 196 by Hakim and Adams [1]. They identified three symptoms that are now recognized as NPH-related: gait impairment, urinary incontinence, and dementia. This condition can be congenital or acquired and affects individuals of all ages, from neonates to the elderly. The actual etiology of NPH remains unknown. NPH is primarily divided into two types: idiopathic NPH (iNPH) and secondary NPH. The most prevalent kind is iNPH, which develops in people with no known secondary cause. Secondary NPH, on the other hand, can develop as a result of cerebral hemorrhage, infection, or other factors that are thought to disrupt the CSF drainage pathways through an inflammatory response, resulting in scarring and/or obstruction, followed by CSF buildup [2].

Many writers have already detailed ways to define hydrocephalus, resulting in distinct definitions [3,4,5,6]. Despite the significant progress over the past century, the classification of Walter Dandy, which was the first one to be developed, remains highly relevant, as its fundamental principles continue to guide treatment decisions. His concept serves as the foundation for determining whether hydrocephalus should be managed with a shunt in communicating cases or through endoscopic fenestration to restore CSF flow between internal and external spaces in non-communicating hydrocephalus [3]. Shizuo Oi proposed a classification of hydrocephalus that focuses on pathophysiological mechanisms and clinical implications. This classification provided a more dynamic perspective on hydrocephalus progression and management, complementing traditional anatomical classifications [6]. Other key advancements in the understanding of hydrocephalus include Russel’s “bulk flow” concept [7], Di Rocco’s theory of “pulse pressure”-induced hydrocephalus [8], and Rekate’s hydraulic circuit model [5]. Despite the advances in diagnosis and treatment, hydrocephalus remains a significant clinical challenge, often requiring lifelong management.

Hydrocephalus can be caused by a variety of factors, including pathophysiologic processes that disrupt CSF flow [9]. CSF, produced by the choroid plexus, flows through the ventricular system and is absorbed by arachnoid granulations into the venous sinuses. With a daily turnover of 500 mL, any obstruction, overproduction, or impaired absorption in its pathway can lead to hydrocephalus [10]. According to the Monro–Kellie doctrine, increased CSF volume raises intracranial pressure, causing brain damage and atrophy [11].

From an anatomical and physiological perspective, understanding the ventricular system, CSF flow dynamics, and the role of the blood–brain barrier is crucial for elucidating the mechanisms of hydrocephalus. Elevated inflammatory cytokines have been detected in both the serum and CSF of patients with hydrocephalus [12,13], and histological analyses reveal neuroinflammatory features, including the activation of macrophages and microglia [14]. Moreover, emerging research in molecular pathways highlights the involvement of neuroinflammatory molecules and aquaporins in the pathogenesis of hydrocephalus. Furthermore, anti-inflammatory treatments have been shown to lower the risk of hydrocephalus in clinical settings and reduce the prevalence of ventriculomegaly in preclinical studies [15]. Genetic factors are increasingly recognized in hydrocephalus pathogenesis, with numerous loci identified in animal models despite limited human research [16]. Hydrocephalus involves molecular abnormalities in brain development, ependymal dysfunction, apoptosis and inflammation, oxidative stress, and recently, impaired glymphatic drainage, which has become a key focus in CSF circulation disorders [16]. Recent research advancements in the genetic and molecular mechanisms of hydrocephalus have facilitated the development of targeted drugs, showing promising results in preclinical studies [17,18].

This article aims to comprehensively review the current molecular and neuroimaging biomarkers associated with hydrocephalus and to provide a state-of-the-art synthesis of the emerging diagnostic and therapeutic strategies. To achieve this, we conducted a narrative review of the existing research and theoretical studies to highlight the available evidence on the relevant biomarkers and to clarify their significance in diagnosis, differentiation from other neurological conditions, potential prognostic value, and implications in targeted intervention.

## 2. Materials and Methods

### 2.1. Search Strategy

This review was conducted by extensively analyzing and synthesizing the current literature related to hydrocephalus, with a specific emphasis on the molecular and neuroimaging biomarkers involved in its pathogenesis, diagnosis, and monitoring. To gather relevant data, a comprehensive search was carried out across major biomedical databases such as PubMed, Scopus, Web of Science, and Google Scholar. The search terms included combinations of “hydrocephalus” and “molecular biomarkers” or “neuroimaging markers” or “diagnostic imaging in hydrocephalus” or “blood biomarkers” or “CSF biomarkers” or “neuroinflammatory biomarkers”. The selection focused on studies addressing both established and emerging biomarkers, aiming to provide an integrated overview of their diagnostic and prognostic relevance. Boolean operators (“AND”, “OR”) were applied to optimize the search strategy. Furthermore, titles and abstracts were screened for synonyms and related terms to ensure the comprehensive coverage of relevant studies.

### 2.2. Eligibility Criteria

The inclusion criteria encompassed peer-reviewed original research articles, systematic reviews, and meta-analyses published in English. Eligible studies focused on molecular and neuroimaging biomarkers relevant to hydrocephalus, including both congenital and acquired forms, and covered various age groups. The exclusion criteria included conference abstracts and studies lacking relevant molecular, imaging, or mechanistic data.

Our primary aim was to discuss all emerging biomarkers relevant to the diagnosis, monitoring, and pathophysiological understanding of hydrocephalus. However, given the broad spectrum of markers investigated over time and the evolving nature of biomarker research in this field, we also reviewed and discussed earlier studies and more established markers that remain important for clinical practice and research.

### 2.3. Data Extraction and Analysis

The data extraction emphasized the key elements such as neuroimaging techniques used in diagnosis and monitoring, molecular biomarkers involved in disease progression or prognosis, as well as relevant changes in CSF dynamics and hydrocephalus pathophysiology. Preclinical studies, both animal models and in vitro investigations, were also reviewed when they contributed meaningful mechanistic understanding.

The findings were critically appraised and synthesized into a comprehensive narrative that highlights the current advances in the identification and clinical utility of biomarkers. The discussion integrates neuroimaging and molecular aspects, with special attention to diagnostic differentiation, monitoring, and potential therapeutic implications in hydrocephalus.

## 3. Molecular Markers

**Figure 1:**
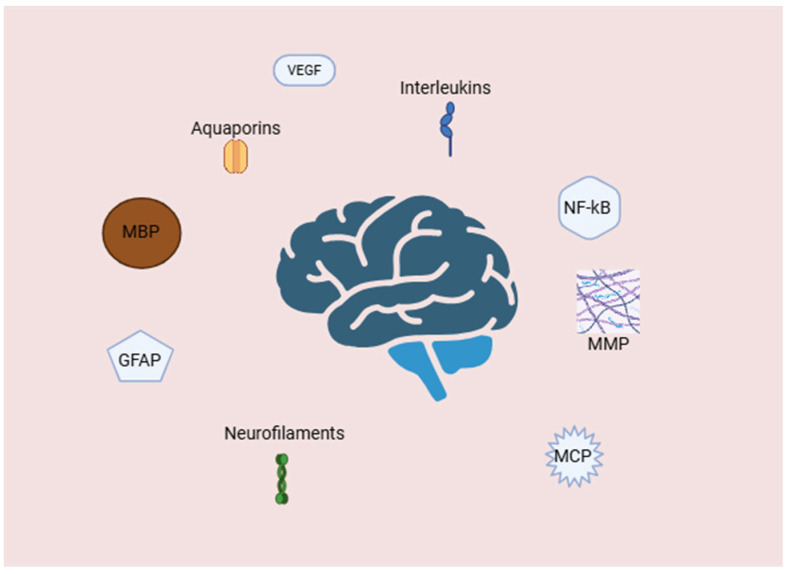
Molecular and cellular mediators of neuroinflammation and blood–brain barrier disruption in hydrocephalus. Abbreviations: GFAP—glial fibrillary acidic protein; MBP—myelin basic protein; MCP—monocyte chemoattractant protein; MMPs—matrix metalloproteinases; NF—κB-nuclear factor kappa-light-chain-enhancer of activated B cells; VEGF—vascular endothelial growth factor. Created with BioRender.com.

Hydrocephalus is typically characterized as an abnormal buildup of CSF within the brain’s ventricles, which causes gradual enlargement and, in many cases, elevated intracranial pressure. While anatomical causes such as aqueductal stenosis or impaired CSF absorption have long been thought to be the primary contributors, new research has revealed the role of the complex molecular and cellular mechanisms in the pathogenesis of both congenital and acquired hydrocephalus, with several markers being studied in the last decades (Figure 1).

### 3.1. Ciliogenesis-Related Genetic Biomarkers

Various hydrocephalus phenotypes appear to converge on a shared pathophysiological mechanism—the disruption of ependymal cilia function, which impairs CSF dynamics and contributes to ventricular enlargement. In the context of biomarker research, several genes have been implicated in the regulation of ciliary structure or motility, suggesting that genetic alterations may serve as potential biomarkers for specific subtypes of hydrocephalus. These biomarkers could not only enhance diagnostic precision but may also open the door to future interventions in the context of personalized medicine [19].

The disruption of ependymal cell function, particularly through impaired ciliogenesis, is a central mechanism contributing to the development of hydrocephalus. Ependymal cilia are essential for CSF circulation, and abnormalities in their structure or function—such as absence, immobility, or misorientation—can impair CSF flow and lead to ventricular enlargement. Several genetic mutations interfere with ciliogenesis by targeting basal bodies (BBs) and centrioles, the foundational structures for cilia formation. Transcription factors such as Forkhead box J1 (FoxJ1), Geminin coiled-coil domain-containing protein 1 (GemC1), and Multiciliate differentiation and DNA synthesis associated cell cycle protein (Mcidas) regulate the differentiation and maturation of multiciliated ependymal cells (MCCs), with deficiencies leading to either complete absence or severe reduction in cilia [20,21,22]. Genes like cyclin O (CCNO), NME/NM23 family member 7 (NME7), and huntingtin (HTT) are also involved in centriole amplification, γ-tubulin ring complex activity, and centrosomal organization, and their disruption can cause failure in cilia development and hydrocephalus [23,24,25]. The structural integrity of the basal bodies is further compromised in models lacking outer dense fiber protein 2 (Odf2) and Na+/H+ exchanger regulatory factor 1 (NHERF1), leading to decreased cilia density or motility, and non-obstructive hydrocephalus [26,27].

Even when cilia form structurally, their inability to beat—due to defects in the dynein arms or axonemal structure—also disrupts CSF dynamics. Dynein arm assembly relies on cytoplasmic preassembly involving dynein axonemal assembly factors (DNAAFs) and co-factors such as leucine-rich repeat-containing protein 6 (LRRC6) and coiled-coil domain-containing protein 151 (CCDC151). Mutations in these genes result in immotile yet structurally preserved cilia [28,29]. The loss of inner dynein arms (IDAs), possibly due to DNA polymerase lambda (DNA Polλ) or indirectly through deletions in primary ciliary dyskinesia homolog (DPCD) mutations, can also halt ciliary motion, although there is still some debate regarding their primary role [30,31]. Moreover, genes such as junctional adhesion molecule-like protein (Jhy) disrupt the “9 + 2” axonemal arrangement essential for motility, leading to nearly immotile “9 + 0” cilia and early-onset hydrocephalus [32]. Finally, proper CSF flow depends not only on the presence and motion of cilia but also their orientation. The disruption of planar cell polarity leads to misdirected ciliary beating, derailing directional CSF flow and contributing further to CSF accumulation and hydrocephalus progression. Thus, the loss, immobility, or disorientation of ependymal cilia—driven by diverse molecular mechanisms—converge on the shared outcome of impaired CSF dynamics and hydrocephalus [19].

### 3.2. Aquaporins

Aquaporins (AQPs), especially AQP1 and AQP4, play a fundamental role in CSF dynamics and have been implicated in the pathophysiology of hydrocephalus. Traditionally, AQP1 has been associated with CSF production due to its expression in the apical membrane of choroid plexus epithelial cells, facilitating water transport into the ventricles [33]. On the other hand, AQP4 is expressed in astrocytic endfeet and ependymal cells, where it contributes to CSF-ISF exchange and drainage through perivascular pathways [34,35]. In animal models, the deletion of either AQP1 or AQP4 leads to reduced CSF formation, and dual knockout significantly impairs CSF outflow and ventricular compliance, reinforcing the notion that both aquaporins contribute significantly and complementarily to CSF homeostasis [36]. These findings suggest that alterations in the expression or localization of these proteins can influence the development and progression of hydrocephalus.

In experimental hydrocephalus models, such as kaolin injection into the cisterna magna, the upregulation of AQP4 has been observed in the hippocampus and cortex, indicating a possible compensatory mechanism aimed at enhancing CSF absorption and mitigating ventricular enlargement [37,38,39]. Conversely, AQP1 expression is often downregulated, with evidence pointing to its intracellular sequestration in choroidal epithelial cells, potentially to limit excessive CSF production and intracranial pressure [40,41]. AQP4 deficiency has been linked to accelerated hydrocephalus progression, while the absence of AQP1 appears protective against ventricular dilation [42]. These observations underscore a dual role for aquaporins—not only as mediators of CSF movement but also as modulators of pathological CSF accumulation—suggesting that abnormal aquaporin expression may either exacerbate or help counteract the hydrocephalic state, depending on the context and cellular localization. New research also demonstrated a progressive, time-dependent upregulation of AQP-9 expression in the CSF of hydrocephalus-induced rats, with statistically significant reductions following CSF drainage [43].

### 3.3. Cytokines, Signaling Pathways, and Neuroinflammatoy Mediators

Cytokine dysregulation plays a critical role in the pathophysiology of hydrocephalus, particularly following hemorrhagic events such as subarachnoid hemorrhage (SAH) and intraventricular hemorrhage (IVH). Proinflammatory and profibrotic cytokines contribute to neuroinflammation, fibrosis, and impaired CSF dynamics, ultimately facilitating the development of chronic hydrocephalus. Among these, transforming growth factor-beta 1 (TGF-β1) has emerged as a key mediator of post-hemorrhagic fibrotic responses [44]. In the central nervous system, TGF-β1 exerts its effects by binding to type I and type II serine/threonine kinase receptors, activating the Smad signaling cascade and promoting extracellular matrix (ECM) deposition and fibroblast-to-myofibroblast transition [45]. Animal models of SAH have demonstrated elevated levels of TGF-β1 in CSF and brain parenchyma, with concomitant increases in Smad2/3 phosphorylation and connective tissue growth factor expression, a profibrotic downstream effector of TGF-β1 [46]. The cytokine displays a biphasic expression pattern in the CSF: an early peak attributed to platelet degranulation, followed by a later phase involving endogenous production by activated CNS cells [47]. The pharmacological inhibition of TGF-β1 signaling using agents such as the Leu-Ser-Lys-Leu (LSKL) peptide or Decorin has shown promise in reducing subarachnoid fibrosis and preventing chronic hydrocephalus in experimental models [48,49]. Nonetheless, conflicting data exist, as older studies report that TGF-β pathway inhibition does not prevent ventricular enlargement in intraventricular hemorrhage (IVH) models [50]. Overall, targeting TGF-β1 and its downstream effectors represents a compelling, though complex, therapeutic avenue in the management of hemorrhage-induced hydrocephalus.

The nuclear factor kappa-light-chain-enhancer of the activated B cell (NF-κB) pathway plays a critical role in neuroinflammation and has been increasingly implicated in the pathogenesis of hydrocephalus. The activation of NF-κB in astrocytes or microglia can trigger the release of pro-inflammatory cytokines, which may disrupt CSF dynamics, impair ependymal cell function, and contribute to ventricular enlargement. In post- IVH hydrocephalus models, the activation of the NF-κB pathway contributes to inflammation, fibrosis, and ventriculomegaly. Inhibiting this pathway with agents such as TAK-242 (a Toll-like receptor 4 (TLR4) inhibitor) or metformin (via the AMP-activated protein kinase/sirtuin 1/NF-κB (AMPK/SIRT1/NF-κB) axis) has been shown to reduce these pathological changes [51,52,53]. Furthermore, the aggregation of choroid plexus (ChP) macrophages can amplify NF-κB signaling in epithelial cells through the tumor necrosis factor alpha/tumor necrosis factor receptor 1 (TNF-α/TNFR1) pathway, promoting excessive cerebrospinal fluid (CSF) secretion and worsening hydrocephalus. Astrocyte-specific activation of the NF-κB pathway, via the constitutively active inhibitor of nuclear factor kappa-B kinase subunit beta (IKK2), also known as IKK2-CA for the constitutively active form), has been shown to cause postnatal hydrocephalus in mice. In a model, transgene expression driven by the glial fibrillary acidic protein (GFAP) promoter leads to ventricular enlargement and hippocampal abnormalities, which are prevented by suppressing IKK2-CA expression with doxycycline. This effect is mediated through canonical NF-κB signaling in astrocytes, highlighting its key role in hydrocephalus pathogenesis [54,55]. Inflammatory markers such as IL-6 and IL-1β are elevated during CNS inflammation and can activate the NF-κB pathway [56,57], which is currently the only demonstrated mechanism—besides malignancy—linked to choroidal CSF hypersecretion and hydrocephalus development [55,58]. IL-18 and vascular endothelial growth factor (VEGF), which also activate NF-κB [59,60,61], may contribute similarly, with VEGF shown to disrupt ependymal integrity and promote ventriculomegaly in rats [62,63]. LRG, induced by IL-6 and IL-1β [64,65], may modulate TGF-β signaling and potentially intersect with NF-κB activity [66,67]. IFN-γ, although primarily signaling via JAK/STAT, also activates NF-κB [68], reinforcing the potential of multiple cytokines to drive CSF hypersecretion. However, inflammation may also promote hydrocephalus through fibrosis and impaired CSF reabsorption, independent of hypersecretion [69,70]. A systematic review discussed inflammatory markers’ changes in hydrocephalus and reported that IL-6, IL-1β, and LRG were most consistently elevated in CSF from iNPH patients, while other markers showed limited or no consistent changes. Also, IL-6, IL-18, and VEGF were most consistently elevated in CSF from post-hemorrhagic hydrocephalus patients, alongside limited increases in 16 other markers and limited decreases in tissue inhibitor of metalloproteinases-4 (TIMP-4) and X-C motif chemokine ligand 1 (XCL-1) [13].

Monocyte Chemoattractant Protein-1 (MCP-1), also known as CCL2, is a chemokine involved in recruiting monocytes and other immune cells to sites of inflammation. Elevated CSF levels of MCP-1 in iNPH, compared to healthy controls or patients with other neurological disorders, suggest its potential role as a biomarker of glial activation and chronic low-grade inflammation in hydrocephalus. Jeppsson et al. found that the levels of MCP-1 might separate iNPH from cognitive and movement disorders [71]. Another study of the same research group showed higher levels of myelin basic protein (MBP), which is a structural component of the myelin sheath, in the CSF of iNPH patients [72].

The markers of neuroinflammation in hydrocephalus have the potential to improve the diagnostic accuracy, refine patient classification, and guide treatment development. Because these molecules can represent both disease activity and the underlying mechanisms, they are useful not only as pathology indicators, but also as targets for treatments.

### 3.4. Matrix Metalloproteinases

Few studies have evaluated the CSF levels of matrix metalloproteinases (MMPs), and their inhibitors (TIMPs) have not been previously reported in iNPH, particularly in relation to hydrocephalus or subcortical small vessel disease [73]. Studies indicate that ECM proteins, MMPs, and their substrates increase in iNPH following shunt surgery, suggesting that the disturbed ECM dynamics are restored by the procedure. MMPs, activated under ischemic conditions, contribute to neuroinflammation by disrupting the blood–brain barrier and degrading the ECM components, promoting ECM turnover. TIMPs primarily regulate MMP activity and may have independent biological functions [72].

A study found that mice lacking membrane-type 1 matrix metalloproteinase (MT1-MMP) developed hydrocephalus characterized by dome-shaped skulls, dilated ventricles, corpus callosum agenesis, and astrocyte hypertrophy. The absence of MT1-MMP led to the impaired maturation of ependymal cells and disorganized motile cilia, resulting in an abnormal CSF flow. These defects were associated with the decreased expression of promulticiliogenic genes and hyperactivation of Notch signaling. Inhibiting Notch signaling restored ciliogenesis, suggesting that MT1-MMP is essential for ependymal cell maturation and ciliogenesis through the suppression of Notch signaling during early brain development [74]. Another study investigated the relationship between MMP-9 and hydrocephalus in patients who underwent craniotomy for severe craniocerebral trauma. The researchers found that the serum levels of MMP-9 were significantly elevated in patients with postoperative hydrocephalus compared to control subjects [75]. Harris et al. highlighted, among the cytokines and MMPs analyzed, that CSF concentrations were significantly elevated for the pro-inflammatory cytokines IL-6 and IL-8, the anti-inflammatory cytokine IL-10, as well as MMP-7 and MMP-9 [76].

### 3.5. Vascular Endothelial Growth Factor

VEGF is a major angiogenesis regulator in the CNS, contributing to neurogenesis, neuroprotection, and blood–brain barrier (BBB) maintenance in addition to vascular development [77]. VEGF has been linked to the pathophysiology of ventricular enlargement in the context of hydrocephalus. Animal models of hydrocephalus have shown elevated VEGF levels, which are linked to aberrant vascular remodeling, periventricular edema, and enhanced BBB permeability. By encouraging aberrant angiogenesis and inflammation in the subarachnoid region, VEGF may also alter the dynamics of CSF, which could lead to poor CSF flow or absorption. Its pathogenic involvement is further supported by animal research that demonstrates that exogenous VEGF can cause ventriculomegaly, whereas VEGF inhibition can stop or lessen these alterations.

Heparin-binding EGF-like growth factor (HB-EGF) regulates forebrain development, while VEGF influences cell migration in the rostral migratory stream. Although HB-EGF’s role in hydrocephalus is unclear, mice overexpressing HB-EGF show elevated VEGF, subarachnoid hemorrhage, and ventriculomegaly. In hydrocephalic rats, VEGF infusions induced ventricular enlargement and altered neuroblast migration, effects prevented by VEGF inhibition [78]. Two studies measured CSF VEGF using the same method but in different age groups. In elderly patients with NPH, VEGF increased post-exercise [79]. In pediatric hydrocephalus, VEGF was higher than in controls, and VEGF-A165 infusion caused ventricular enlargement, accompanied by increased VEGFR2 phosphorylation in the ependyma, changes in β-catenin and E-cadherin levels, ependymal denudation, and disrupted ciliary staining [62]. The extent of ventricular dilation varies with the infusion rate and duration [62]. VEGF primarily signals through VEGFR2, triggering receptor dimerization and Src kinase activation. This process promotes VE-cadherin phosphorylation, internalization, and reduced binding to p120- and β-catenin, weakening cell junctions [80,81,82]. As VE-cadherin relocates from the membrane to the cytoplasm, junctional integrity is lost, BBB permeability increases, and hydrocephalus may develop [83]. Huang et al. linked elevated VEGF levels post-exercise to distinct CSF metabolomic shifts in patients with NPH, implicating VEGF as a potential modulator of brain energy metabolism and methylation pathways in the context of hydrocephalus pathophysiology [84].

### 3.6. Neurofilaments

Neurofilaments, especially the neurofilament light chain (NfL), have shown promise as indicators of neuronal damage in a variety of neurological diseases, including hydrocephalus [85]. In the setting of hydrocephalus, excessive levels of NfL in CSF or blood indicate axonal damage caused by increased intracranial pressure, ventriculomegaly, or poor CSF dynamics.

Their value stems from the fact that they provide a measurable measure of neurological injury that can be used to aid in diagnosis, monitor disease development, and even assess the therapy response, such as after shunt insertion. According to studies, patients with iNPH had greater CSF NfL levels than healthy controls [86,87,88]. However, Jeppsson et al. found that the levels of NFL did not differ between iNPH patients and healthy controls [89]. NFL may nowadays be detected in plasma, allowing axonal degeneration to be monitored without the need for a lumbar puncture [90]. NFL plasma has been effectively evaluated in various neurological disorders, such as Alzheimer’s disease, Parkinson’s disease, and HIV-associated dementia [91,92,93], and it could pave the way for a less invasive biomarker sample for disease diagnosis and monitoring.

### 3.7. Glial Fibrillary Acidic Protein

Glial fibrillary acidic protein (GFAP) is an intermediate filament protein found in astrocytes that is commonly used as a marker of astroglial activity or damage. Elevated GFAP levels associated with hydrocephalus indicate reactive astrogliosis, which occurs in reaction to mechanical stress and damage produced by ventricular enlargement and abnormal CSF dynamics [94]. Increased GFAP concentrations have been observed in older studies in hydrocephalus [95], while Tang et al. reported a case which showed positive anti-GFAP-IgG antibodies in serum and CSF [96]. Nevertheless, another recent investigation that evaluated GFAP concentrations in NPH patients versus individuals with subcortical arteriosclerotic encephalopathy found that there was no significant difference between the two groups [72].

Table 1 presents a summary of biomarkers implicated in the pathophysiology of hydrocephalus, outlining their altered expression patterns and associated biological effects.

### 3.8. Proteomic Markers

Beyond traditional imaging and genetic methods, proteomic analyses of CSF and plasma have started to identify new biomarkers that could provide further understanding of the pathogenesis of hydrocephalus. According to studies that found quantitative proteomics markers in post-hemorrhagic and idiopathic NPH, TLR4-NF-κB, mTOR, PDGFRα signaling, and other pathways may be particularly relevant [55,110,111]. Early candidate studies demonstrated alterations in acute-phase and transport proteins, such as clusterin, kininogen, transthyretin, β2-microglobulin, retinol-binding protein, and apolipoproteins A1, A4, and E, in CSF from patients with NPH compared to controls, suggesting disruptions in protein folding, lipid transport, and blood–brain barrier integrity [94,112,113]. More recent large-scale CSF proteomic screens using mass spectrometry have identified the upregulation of complement components, matrix metalloproteinases, and acute-phase reactants, implicating immune activation, extracellular matrix remodeling, and neuroinflammation in ventricular enlargement and white matter injury [106,114,115]. Although less well studied in hydrocephalus, plasma proteomic signatures in other neurological diseases show elevated levels of fibrinogen, complement factors, acute-phase reactants and interleukins, suggesting systemic inflammatory and vascular contributions to disease pathophysiology [116,117]. Integrating these proteomic discoveries with neuroimaging and genetic data offers a more comprehensive biomarker model for hydrocephalus: clusters of inflammatory and proteolytic mediators in CSF could predict a shunt response, but plasma-derived panels could allow for the early detection and monitoring of disease development. However, prior to clinical adoption, these putative biomarkers must be validated in larger, multicenter cohorts and standardized across analytical platforms.

## 4. Neuroimaging Techniques and Markers

Neuroimaging biomarkers in hydrocephalus are critical for the proper diagnosis, treatment planning, and monitoring of the response to therapy. They provide objective, quantitative data into anatomical and functional brain alterations, which aid in distinguishing hydrocephalus from other neurological diseases. This is especially essential in the differential diagnosis since hydrocephalus symptoms, such as cognitive impairment, gait disturbances, or incontinence, might be similar to those of neurological conditions like Alzheimer’s or Parkinson’s. Many studies have discussed imaging markers in the context of differentiating hydrocephalus from other neurological diseases (Table 2).

**Figure 2:**
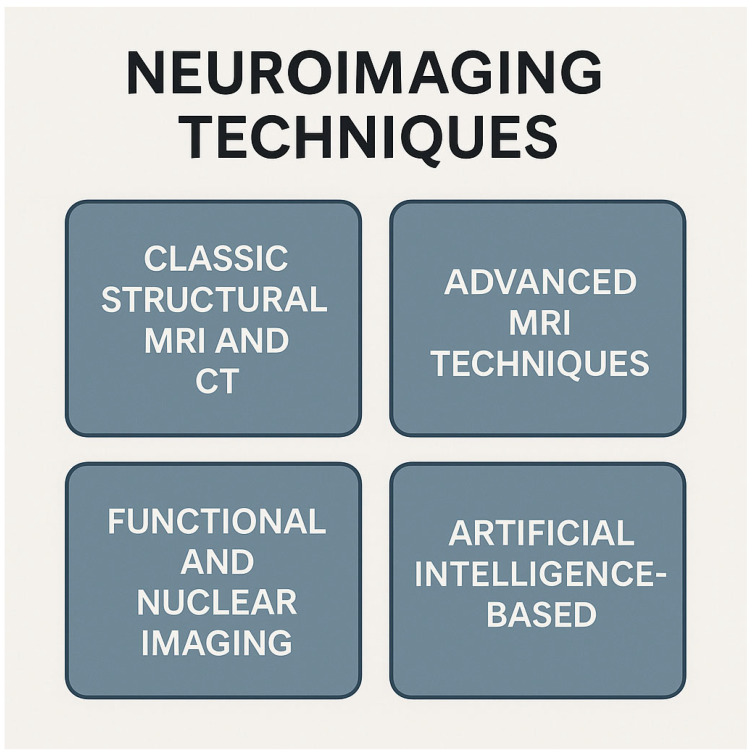
Classification of neuroimaging techniques into four categories. Conventional structural MRI and CT provide detailed anatomic images; advanced MRI methods offer microstructural and hemodynamic insights; functional and nuclear imaging map brain activity, metabolism, and molecular targets; and artificial intelligence-based approaches are used for image analysis, lesion detection, and predictive modeling. Abbreviations: CT—computer tomography; MRI—magnetic resonance imaging. Created with Biorender.com.

Recent advances in magnetic resonance imaging (MRI) and computed tomography (CT) have led to several specialized techniques for assessing hydrocephalus (Figure 2). Together, these modalities enhance the diagnostic accuracy, aid in distinguishing hydrocephalus subtypes, and provide valuable biomarkers for treatment planning and follow-up.

### 4.1. Classic Structural MRI and CT

When diagnosing and evaluating hydrocephalus, conventional radiological indicators are essential. These indicators aid in differentiating hydrocephalus from other causes of ventriculomegaly and are commonly detected on non-contrast CT or MRI scans. Since CT has a rapid acquisition time, is widely available, and can clearly show ventricular enlargement, it is essential for the diagnosis and assessment of hydrocephalus. It is especially helpful in emergency circumstances where the early identification of ventricular dilatation, periventricular edema, or mass effect is crucial, such as suspected obstructive hydrocephalus or trauma-related instances.

**Figure 3:**
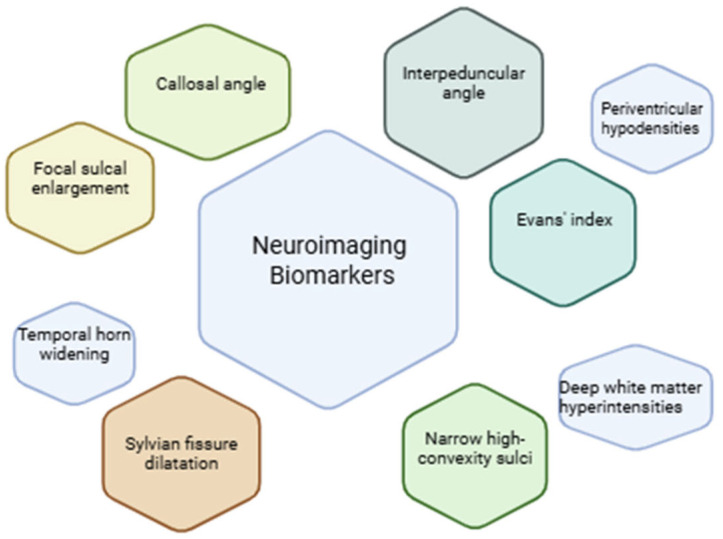
Common neuroimaging biomarkers evaluated with CT and MRI in the diagnosis of hydrocephalus.

However, the lack of standardized imaging criteria makes identifying NPH difficult [134]. Evans’ index, narrower high-convexity sulci, Sylvian fissure dilatation, focal sulcal enlargement, temporal horn widening, callosal angle, and periventricular hypodensities are examples of common CT-based metrics [135] (Figure 3)

MRI is a safer alternative and relies on strong magnetic fields and radio waves, offering detailed brain imaging without radiation. Over the past two decades, in vivo MRI and spectroscopy have become key tools for analyzing brain function. MRI detects more lesions than CT and provides consistent imaging even with repeated scans. It works by aligning hydrogen protons in tissues, which resonate under radiofrequency pulses, typically using 0.5–1.5 tesla field strengths. MRI effectively distinguishes normal from abnormal brain tissue and is especially valuable for studying hydrocephalus, particularly changes in the periventricular regions [136].

MRI generally requires more time or more resources and is more expensive to operate than CT. Furthermore, because standard MRI sequences are sensitive to patient movement, sedation or anesthesia is frequently required for young and/or uncooperative children [137]. Researchers may evaluate several anatomical biomarkers, one of the most widely used metrics being Evans’ index, which is determined by dividing the maximum internal diameter of the skull by the maximum width of the frontal horns of the lateral ventricles. Generally speaking, hydrocephalus is suggested by an Evans’ index higher than 0.3. A narrowed callosal angle (less than 90°), which is measured on coronal imaging through the posterior commissure, is linked to NPH and aids in distinguishing it from cerebral atrophy, where the angle is typically broader [138].

Neurodegenerative changes were assessed using Scheltens’ score for medial temporal atrophy (MTA), the Fazekas scale for white matter lesions, and entorhinal cortex (ERC) thickness [139]. Eide et al. showed that in patients with iNPH, MRI biomarkers revealed significantly slower clearance of CSF tracer from both the cisterna magna and the entorhinal cortex [140]. Another study utilized several imaging biomarkers to differentiate iNPH from progressive supranuclear palsy, including the hummingbird sign indicative of midbrain tegmental atrophy, disproportionately enlarged subarachnoid space hydrocephalus (DESH), magnetic resonance parkinsonism index (MRPI), Evans’ index, callosal angle, magnetic resonance hydrocephalic index (MRHI), which represented a novel biomarker developed by the authors, and automated ventricular volumetry (AVV). They highlighted that the most reliable imaging biomarkers were AVV and MRHI [132]. Periventricular hyperintensities have also been employed as an iNPH MRI marker and represent T2-hyperintense white matter lesions adjacent to the frontal and occipital horns of the lateral ventricles which are relatively common in the elderly [141]. The imaging pattern known as DESH score is an integrated neuroimaging index composed of five markers: ventriculomegaly, high tight convexity, dilated Sylvian fissures, acute callosal angle, and focal sulcal dilatation [142]. DESH is seen as a feature of NPH [128,140,143]. If the lateral ventricles’ temporal horns dilate more than their overall ventricular expansion, it may indicate hydrocephalus and be a precursor to elevated intracranial pressure. Periventricular hyperintensities or hypodensities indicate elevated intraventricular pressure and reflect transependymal CSF flow; they frequently coexist with obstructive or decompensated hydrocephalus. A further supporting characteristic on imaging is the corpus callosum’s thinning or elevation, which can be caused by persistent ventricular dilatation [138].

MRHI also demonstrated excellent diagnostic accuracy in distinguishing iNPH from progressive supranuclear palsy, outperforming callosal angle measurements and misclassifying only a small number of cases in recent studies [132]. Kockum et al. proposed the Radscale, a structured radiological tool for evaluating iNPH, which scores seven features: Evans’ index, callosal angle, focal and narrow sulci, dilation of the temporal horns and Sylvian fissures, and periventricular signal changes [135]. Most are graded 0–2, except focal sulci and Sylvian fissure dilation, which are scored 0 or 1 [135].

### 4.2. Machine Learning and Artificial Intelligence

As technology has advanced, artificial intelligence (AI) has become a useful tool in radiology, providing increased diagnosis accuracy through the quick analysis of imaging data and the detection of small anomalies. The detection of NPH-related characteristics can be improved by using deep learning models, which can recognize intricate patterns in CT and MRI scans [144].

A common AI-based diagnostic pathway for NPH includes the following: delineation of the CSF and ventricles in MRI/CT scans, collection of critical volumetric data, and categorization using machine learning algorithms to differentiate NPH from non-NPH cases [145,146]. In one recent study, an AI tool was developed to process DICOM-format brain CT images, automatically identify CSF and brain tissue, extract radiological features like ventricular shape and Evans’ index, and integrate these with patient age to train a neural network using transfer learning techniques. The resulting AI model achieved a diagnostic accuracy of 94.0%, with a sensitivity of 93.6% and specificity of 94.4% (AUC: 0.93) [145]. Prevedello et al. [15] also reported that their AI model for diagnosing hydrocephalus achieved an accuracy of up to 90% [147]. A systematic review evaluated AI-based models and evaluated the differences between traditional machine learning (such as Random Forest and Logistic Regression), deep learning, and hybrid approaches for NPH diagnoses. Traditional machine learning (ML) methods showed accuracies between 70% and 96% in classifying NPH, with the best results (96.3%) using Logistic Regression to distinguish NPH from Alzheimer’s disease. These models offer interpretable outputs but rely heavily on manual feature selection. In contrast, deep learning models achieved higher accuracies (90–99.1%), with automatic feature extraction and better pattern recognition, though they require large datasets and high computational power, and often lack interpretability, posing challenges for clinical use [148]. Fernandes et al. conducted a systematic review to assess the efficacy of AI in predicting the shunt response in iNPH, with only four studies reporting AUC values (between 0.80 and 0.94). They also emphasized the variability in outcomes, data heterogeneity, and possible model bias [149].

### 4.3. Functional and Nuclear Imaging

An extensive review discussing Positron Emission Tomography (PET)-CT concluded that, in 20–57% of patients, amyloid PET identifies concurrent Alzheimer’s pathology, which may help predict surgical prognosis. Global and basal ganglia-specific cerebral blood flow (CBF) loss is seen via perfusion PET, and preoperative perfusion parameters are correlated with the postoperative results. Postoperative striatal D2 receptor upregulation and postsynaptic D2 receptor loss are indicated via dopaminergic PET and are also associated with clinical recovery [150]. Additionally, PET imaging may identify distinctive patterns of hypometabolism in NPH individuals; however, its clinical value remains limited [151]. In patients with iNPH, Fluorodeoxyglucose (FDG) PET/CT imaging—through voxel-wise analysis using SSM-PCA—can identify a characteristic metabolic spatial covariance pattern (iNPHRP) that differentiates iNPH from other neurodegenerative conditions such as Alzheimer’s and Parkinson’s disease with high diagnostic accuracy (up to 100%) [152].

Dopamine Transporter Single-Photon Emission Computed Tomography (DAT-SPECT) is a neuroimaging technique that assesses the integrity of dopaminergic neurons by visualizing the dopamine transporter availability in the striatum. Studies have shown reduced striatal DAT binding in iNPH, which may reflect either primary dopaminergic degeneration or secondary effects from disrupted CSF flow and ventricular enlargement. DAT-SPECT can aid in distinguishing iNPH from neurodegenerative parkinsonian syndromes [153] and may help predict motor improvement following CSF shunting. However, a study by Ma et al. evaluated the role of DaT-SPECT in predicting the response to ventriculoperitoneal shunting in NPH. Their findings suggest that while DaT-SPECT is useful in diagnosing parkinsonian syndromes, it has limited value in guiding management decisions in NPH, as dopaminergic dysfunction may coexist with shunt-responsive NPH [154].

### 4.4. Advanced MRI Techniques

MRI elastography (MRE) is a non-invasive imaging method that visualizes the propagation of mechanical shear waves through tissue to quantify mechanical characteristics including elasticity and stiffness. MRE can identify minor changes in viscoelasticity in the brain that are linked to tumors, hydrocephalus, neurodegenerative diseases, or aging [155,156]. INPH has been linked to reduced brain stiffness, especially in the periventricular white matter and basal ganglia [157,158]. This may be correlated with clinical symptoms and predict the results of surgery [157,159]. Fattahi et al. demonstrated that, in contrast to healthy controls, patients with NPH showed increased stiffness in the occipital, parietal, and temporal lobes and lowered elasticity in the frontal lobe and deep gray matter/white matter (GM/WM) regions [160]. By offering functional biomechanical information, MRE supplements conventional structural MRI and may improve the treatment planning and diagnostic precision for a range of neurological disorders.

Diffusion tensor imaging (DTI) is an advanced MRI technology that provides information on the microstructural integrity of the brain by mapping the diffusion of water molecules along white matter tracts. It is especially helpful when evaluating white matter injury or disarray in neurological conditions. In iNPH, DTI has shown decreased fractional anisotropy and increased mean diffusivity in periventricular regions, especially the corpus callosum and corona radiata, which are indicative of demyelination or axonal injury [161,162,163]. Grazzini et al. employed DTI to assess white matter microstructural changes in patients with iNPH. The technique revealed significantly reduced fractional anisotropy and increased mean diffusivity and axial diffusivity (AD) in several white matter tracts, indicating disrupted fiber integrity [164]. In addition to being possible biomarkers for predicting shunt responsiveness and tracking treatment outcomes [165], these DTI changes may help distinguish iNPH from other neurodegenerative diseases [166].

The noninvasive imaging method known as arterial spin labeling (ASL) MRI measures CBF without the need for contrast agents or radiation exposure. ASL MRI has shown decreased perfusion in areas such as the thalamus, basal ganglia, and periventricular white matter in patients with iNPH in comparison to healthy controls. Furthermore, a lower CBF in these regions is associated with greater cognitive impairment, indicating that ASL MRI may be a useful diagnostic and severity assessment technique for iNPH. Virhammar et al. revealed significantly lower CBF in areas such as the periventricular white matter, lentiform nucleus, and thalamus compared to controls. ASL helped identify vascular artifacts in some patients, but CBF differences remained significant after excluding them. The technique was also found to correlate with clinical symptoms, such as the mini-mental state examination (MMSE) score, particularly in regions like the pons and cerebellum [167]. The degree of ventricular dilation is also correlated with CBF regulation in specific brain regions, especially the watershed areas [168]. ASL MRI is a potential technique for assessing individuals with suspected hydrocephalus because of its noninvasive nature and capacity to identify perfusion abnormalities. Bagatto et al. found that ASL-MRI could noninvasively detect perfusion changes and may help identify which patients are likely to benefit from surgery [169].

### 4.5. Glympathic MRI

Glymphatic MRI is a promising imaging technique to study CSF dynamics and waste clearance in the brain, which is crucial in neurological diseases such as Alzheimer’s disease, hydrocephalus, and brain trauma. This study explored the use of gadobutrol contrast enhancement in patients with iNPH using glymphatic MRI. The researchers found delayed gadobutrol distribution in iNPH patients, particularly in the subarachnoid spaces and ventricles, indicating disrupted CSF dynamics. Enhanced gadobutrol uptake in brain parenchyma, especially in areas like the frontal horn and inferior frontal gyrus, suggested impaired clearance [170]. A research group performed numerous experiments in which contrast agents were given intrathecally to both patients with NPH and a healthy cohort [139,144,171,172]. In one of them, MRI biomarkers have been suggested using a ventricular reflux grade system, showing both the impairment of glymphatic and meningeal lymphatic systems and suggesting the redirection of CSF flow to the ventricles in individuals with iNPH [141].

## 5. Limitations and Perspectives

New imaging modalities and fluid biomarkers hold the promise of the earlier, more precise characterization of hydrocephalus subtypes: advanced MRI sequences such as diffusion tensor imaging and phase-contrast flow studies, when combined with cerebrospinal fluid or blood markers such as neurofilament light chain or aquaporin-4 autoantibodies, could allow for the noninvasive phenotyping and real-time monitoring of disease progression and treatment response. When combined with machine learning analyses of imaging, genetic, and clinical data, these tools might lead the way for truly personalized therapeutic strategies, such as guiding decisions between shunting and endoscopic third ventriculostomy, optimizing programmable drainage valves, and even directing targeted molecular interventions designed to regulate ependymal ciliary function or CSF production.

However, the heterogeneity of hydrocephalus etiologies must be taken into consideration. Congenital, post-hemorrhagic, tumor-associated, and idiopathic normal-pressure variants all follow different pathophysiological pathways, making it more difficult to design treatments that are generally effective and to interpret the results of clinical trials. The generalizability of preclinical results is limited by the fact that animal models, while useful for mechanistic knowledge, frequently do not replicate human CSF dynamics and immune responses. The lack of large prospective longitudinal cohorts with standardized outcome measures makes it difficult to evaluate the long-term neurocognitive consequences, optimize shunt settings, and schedule updates using data. Furthermore, invasive sampling for novel biomarkers raises ethical issues, especially in pediatric populations, and advanced technology such as advanced endoscopic equipment or telemetric shunt monitoring are still unattainable in many low-resource settings. Last but not least, despite their theoretical appeal, regenerative and gene-based therapies are delayed from bench to bedside by significant obstacles related to immunological compatibility, long-term safety, and transport over the epidermal barrier. Despite these obstacles, if interdisciplinary efforts can match cutting-edge technologies with thorough clinical validation, the sector is set for revolutionary advancement.

## 6. Conclusions

Hydrocephalus is now understood to involve far more than just mechanical obstruction or impaired CSF absorption. Recent advances in molecular biology have revealed that a complex relationship between neuroinflammation, genetic predispositions affecting ependymal integrity and glymphatic clearance, and dysregulated aquaporin channels all contribute to ventricular enlargement and periventricular injury. At the same time, neuroimaging techniques have become indispensable for distinguishing iNPH from other causes of gait disturbance, cognitive decline, or bladder dysfunction. Moreover, combining fluid biomarkers with imaging signatures holds great promise for a truly multimodal approach, in which molecular profiles guide imaging interpretation and vice versa. This convergence of laboratory and radiological insights marks a shift toward earlier, more precise diagnosis and personalized therapeutic strategies, potentially improving outcomes by identifying high-risk patients before irreversible brain injury occurs.

## Figures and Tables

**Table 1:** Summary of key biomarkers associated with hydrocephalus, their expression changes, and functional implications.

Biomarker	Observed Changes/Associations	Study
AQP1	Decreased expression, intracellular sequestration; possibly limits CSF production	Baktir et al. [97]
AQP4	Increased expression; may enhance CSF absorption	Bloch et al. [38,42], Verkman et al. [98]
AQP9	Time-dependent increase; reduced after CSF drainage	Kusumo et al. [43]
TGF-β1	Elevated post-hemorrhage; promotes fibrosis via the Smad pathway	Zhan et al. [99]
IL6	Elevated in iNPH and post-hemorrhagic hydrocephalus; activates NF-κB	Lolansen et al. [13], Mehmedika-Suljić et al. [100]
IL-1β	Elevated in iNPH; activates NF-κB	Lolansen et al. [13], Mehmedika-Suljić et al. [100]
IL18	Elevated post-hemorrhage; activates NF-κB	Lolansen et al. [101], Schmitz et al. [102]
VEGF	Elevated in hydrocephalus; disrupts ependymal cells; promotes ventriculomegaly	Shim et al. [62]
LRG	Elevated in iNPH; modulates TGF-Î^2^ and intersects with NF-κB	Lolansen et al. [13]
IFN	Elevated; activates NF-ÎºB; contributes to inflammation	Lolansen et al. [13]
MCP-1	Elevated in iNPH; suggests glial activation and chronic inflammation	Braun et al. [103], Yang et al. [104].
MBP	Elevated in iNPH; marker of myelin damage	Kaya et al. [105]
MMP-7	Elevated post-trauma; involved in ECM turnover	Harris et al. [76]
MMP-9	Elevated in post-trauma and inflammation; associated with ventriculomegaly	Okamoto et al. [106]
MT1-MMP	Deficiency leads to hydrocephalus; essential for ependymal maturation	Jiang et al. [74]
CXCL-1	Limited decrease in post-hemorrhagic hydrocephalus	Habiyaremye et al. [107]
NfL	Elevated in hydrocephalus; indicates axonal damage	Kaya et al. [105]
TIMP-4	Reported limited decrease in post-hemorrhagic hydrocephalus	Killer et al. [108]
GFAP	Increased in reactive astrogliosis; linked to mechanical stress from ventriculomegaly	Kaya et al. [105]
NF-κB	Activated in astrocytes/microglia; induces inflammation, fibrosis, and CSF hypersecretion	Xu et al. [109], Lattke et al. [54].

Abbreviations: AQP—aquaporin; CSF—cerebrospinal fluid; CXCL-1—C-X-C motif chemokine ligand 1; ECM—extracellular matrix; GFAP—glial fibrillary acidic protein; IFN—interferon; IL—interleukin; iNPH—idiopathic normal pressure hydrocephalus; LRG—leucine-rich alpha-2-glycoprotein; MBP—myelin basic protein; MCP-1—monocyte chemoattractant protein-1; MMP—matrix metalloproteinase; MT1-MMP—membrane-type 1 matrix metalloproteinase; NF-κB—nuclear factor kappa-light-chain-enhancer of activated B cells; NfL—neurofilament light chain; TGF-β1—transforming growth factor beta 1; TIMP-4—issue inhibitor of metalloproteinases-4; VEGF—vascular endothelial growth factor.

**Table 2:** Research studies using neuroimaging as a tool in the differential diagnosis of hydrocephalus.

Study	Neuroimaging Marker	Differential Diagnosis	Neuroimaging Method
Mantovani et al. [118]	ACA, CA, Evans’ index	Alzheimer’s disease	MRI
Ishii et al. [119]	CA, Evans’ index	Alzheimer’s disease	MRI
Cagnin et al. [120]	CA (simplified), Evans’ index, parietal/frontal narrowing, empty sella, posterior cingulate narrowing	Dementia with Lewy bodies, Alzheimer’s disease	MRI
Fallmar et al. [121]	Evans’ index, compression of (tight) high-convexity sulci, enlargement of Sylvian fissures, presence of focally enlarged sulci, width of temporal horns, callosal angle, periventricular hyperintensities, deep white matter hyperintensities, and ventricular roof bulgings	Vascular dementia, atypical parkinsonism	MRI
Quattrone et al. [122]	Combination of Evans’ index and Callosal angle, DESH, MRHI, ventricular volume/intracranial volume ratio	Progressive supranuclear palsy.	MRI
Savolainen et al. [123]	Extent and pattern of hippocampal atrophy and peri-hippocampal dilation	Alzheimer’s disease	MRI
Ugga et al. [124]	Interpeduncular angle, MRHI	Progressive supranuclear palsy,	MRI
Caligiuri et al. [125]	Microstructural changes in white matter	Progressive supranuclear palsy, Alzheimer’s disease	DTI
Kang et al. [126]	Microstructural changes in white matter	Alzheimer’s disease	DTI
Kim et al. [127]	Microstructural changes in white matter	Alzheimer’s disease, subcortical vascular dementia	DTI
Horinek et al. [128]	Microstructural changes in white matter	Alzheimer’s disease	DTI
Ivkovic et al. [129]	Microstructural changes in white matter	Dementia with Lewy bodies, Alzheimer’s disease, Parkinsons’ disease	DTI
Marumoto et al. [130]	Microstructural changes in white matter	Parkinsons’ disease	DTI
Younes et al. [131]	Microstructural changes in white matter	Alzheimer’s disease	DTI
Quattrone et al. [132]	MRHI, automated ventricular volumetry	Progressive supranuclear palsy	MRI
Kuroda et al. [133]	White matter lesion distribution	Alzheimer’s disease	MRI

Abbreviations: ACA—anterior callosal angle; CA—callosal angle; DESH—disproportionately enlarged subarachnoid space hydrocephalus; DTI-diffusion tensor imaging; MRHI—magnetic resonance hydrocephalic index; MRI—magnetic resonance imaging.

## Data Availability

No new data created.
